# Gender equity programmes in academic medicine: a realist evaluation approach to Athena SWAN processes

**DOI:** 10.1136/bmjopen-2016-012090

**Published:** 2016-09-08

**Authors:** Louise Caffrey, David Wyatt, Nina Fudge, Helena Mattingley, Catherine Williamson, Christopher McKevitt

**Affiliations:** 1 Faculty of Life Sciences & Medicine, Division of Health & Social Care Research, King's College London, London, UK; 2 NIHR Biomedical Research Centre at Guy's and St. Thomas’ NHS Foundation Trust and King's College London, UK; 3 Arts & Sciences Faculties, King's College London, London, UK; 4 Women's Health Academic Centre, King's College London, Guy's Hospital, London, UK

**Keywords:** gender equity, Athena SWAN, Realist Evaluation, Academic medicine, gender inequality

## Abstract

**Objectives:**

Gender inequity has persisted in academic medicine. Yet equity is vital for countries to achieve their full potential in terms of translational research and patient benefit. This study sought to understand how the gender equity programme, Athena SWAN, can be enabled and constrained by interactions between the programme and the context it is implemented into, and whether these interactions might produce unintended consequences.

**Design:**

Multimethod qualitative case studies using a realist evaluation approach.

**Setting:**

5 departments from a university medical school hosting a Translational Research Organisation.

**Participants:**

25 hours of observations of gender equality committee meetings, 16 in-depth interviews with Heads of Departments, Committee Leads and key personnel involved in the initiative. 4 focus groups with 15 postdoctoral researchers, lecturers and senior lecturers.

**Results:**

The implementation of Athena SWAN principles was reported to have created social space to address gender inequity and to have highlighted problematic practices to staff. However, a number of factors reduced the programme's potential to impact gender inequity. Gender inequity was reproduced in the programme's enactment as female staff was undertaking a disproportionate amount of Athena SWAN work, with potential negative impacts on individual women's career progression. Early career researchers experienced problems accessing Athena SWAN initiatives. Furthermore, the impact of the programme was perceived to be undermined by wider institutional practices, national policies and societal norms, which are beyond the programme's remit.

**Conclusions:**

Gender equity programmes have the potential to address inequity. However, paradoxically, they can also unintentionally reproduce and reinforce gender inequity through their enactment. Potential programme impacts may be undermined by barriers to staff availing of career development and training initiatives, and by wider institutional practices, national policies and societal norms.

**Strengths and limitations of this study**

- The study investigates the enactment of an internationally adopted gender equity programme (Athena SWAN) in a medical school hosting a Translational Research Organisation.
- Uses a realist evaluation approach to explore how the impact of implementing Athena SWAN principles might be enabled or constrained by interactions between the programme and the context it is introduced into and whether unintended consequences might occur.
- Adopts a multimethod qualitative approach encompassing observations of gender equity committees as well as interviews and focus groups with staff.
- This research was limited to one higher education institute; therefore, future research could add further depth to the themes identified.

## Introduction

Under-representation of women in academia has been internationally identified as a problem to be addressed.^1–8^ While there has been significant progress in the representation of women in clinical medicine, gender imbalances have persisted in academic medicine, particularly at senior levels.^1^ ^9^ In the UK just 28% of clinical academics are women and gender inequality increases with seniority: 42% of lecturers are women but only 18% of professors are women.^10^ International evidence indicates that men are more likely than women to be employed by universities, to start their careers at a higher grade, to receive higher salaries at each academic grade and to reach senior academic levels and hold senior management positions.^6^ ^11^ It has been argued that the under-representation of women is the result of decades of bias, unequal opportunities, gender stereotypes and prejudice.^3^ ^8^ ^12^

From an economic perspective, the under-representation of women in academic medicine wastes public investment^9^ and may constitute a threat to international competitiveness of a country's translational research.^13^ The under-representation of women in academic medicine is also problematic from a participatory/rights perspective since this infringes the democratic principle that citizens should have equity of opportunity to participate in all aspects of society, including work.^6^

The Athena SWAN programme evolved from work between the Athena Project and the SWAN (Scientific Women's Academic Network). The term ‘Athena SWAN’ is now treated as a proper noun, rather than an acronym.^14^ At the time of the data collection, Athena SWAN sought to advance women's careers in Science Technology Engineering Maths and Medicine (STEMM). Since May 2015 it has expanded its remit to focus on gender parity and encompass non-STEMM disciplines.^14^ It was established in the UK in 2005 and has been adopted by other countries, including Ireland and Australia (where it is known as SAGE, ‘Science in Australia Gender Equity’) and there have been discussions about expanding it further, including to Canada, New Zealand, Sweden, Norway and the USA.^15^ ^16^

The Athena SWAN programme offers three awards, Bronze, Silver and Gold, representing different achievements in promoting and documenting gender equity in the practices of HE departments and higher education institutes (HEIs). These awards provide a means for HEIs to document and benchmark their performance in relation to gender equity. Awards are based on the work of self-assessment teams (SATs), which are comprised of members of staff and are set up in HE departments. SATs are tasked with collating and analysing data for the award application. The application focuses on identifying good practice as well as barriers to gender equity, constructing an action plan to address the problems identified, implementing these actions and evaluating outcomes. SATs make applications to Athena SWAN for awards. Throughout this article we distinguish between Athena SWAN in its entirety as a ‘programme’ and the ‘initiatives’ that comprise it, for example, mentoring^17^ and leadership development initiatives^18^ and unconscious bias training.^19^

In 2011 it was announced that UK universities hosting TROs (known in the UK as Biomedical Research Centres) would likely require a Silver Award in order to be eligible to apply for funding to renew TRO contracts.^9^ UK TROs are cohosted by an HEI and a hospital trust. These have separate practices and policies, although many staff work across the three institutions and so are colocated. Despite the implications of Athena SWAN Awards for TRO funding, assessment for awards is focused only on practice in HEIs. This announcement, nonetheless, linked Athena SWAN awards to a major financial incentive.

Research on Athena SWAN has thus far focused on the programme's impact on gender equity. A recent econometric analysis of the impact of Athena SWAN found an increase in the number of women in academic medicine in the UK but no evidence that this was on account of either the introduction of Athena SWAN Awards or the 2011 announcement to tie future TRO funding to Silver Awards.^20^ However, the small number of medical schools in the UK may have constrained the precision of the estimates and the longer term impact of Athena SWAN could not be established at this stage.^20^

Munir *et al*^21^ found that women respondents to a stratified sample of UK HEIs thought that Athena SWAN had improved their visibility, increased their self-confidence, enhanced their leadership skills, helped them to think more broadly about gender issues and had impacted positively on their career development. However, the study suggested less of an impact on research staff and undergraduate students. Participants in this study further reported that linking awards to funding ensured that institutions took them seriously. In keeping with this, Gregory-Smith^20^ found that by 2013, medical schools with a TRO were significantly more likely than those without to have a Bronze award.

Munir *et al*^21^ reported that the workload of Athena SWAN was considered ‘appropriate’ by Athena SWAN Institutional Leads, while Departmental Leads' perceptions were split between ‘excessive’ (49%) and ‘appropriate’ (49%). Little else is known about how the Athena SWAN application process and Athena SWAN initiatives are experienced by staff. Yet this is important. Realist evaluation^22^ suggests that, while programmes may have the capacity for impact, whether they are effective or not depends on their interaction with contextual factors, which can either support or undermine the impact of a programme. In addition, realist evaluation highlights the potential for programmes to produce unintended consequences. The approach, therefore, asserts the importance of examining how programmes interact with contextual factors and with what (unintended) consequences.

In order to address this gap in the literature, this study adopted a novel approach, grounded in the realist evaluation principles referred to above. It explored how Athena SWAN principles were put into practice in five departments in one UK medical school and with what consequences for staff. We sought to understand what was involved in Athena SWAN work and who was actively involved, as well as how the programme was experienced and understood, particularly by postdoctoral researchers and lecturers at the ‘bottlenecks’ within the academic career trajectory.^23^ In order to take account of the potential impact of the linking of funding for TROs, we undertook the study in a medical school hosting a TRO. The findings contribute new and unique knowledge on gender equity initiatives to support women's representation in academic medicine.

## Methods

Drawing on the principles of realist evaluation,^22^ this study sought to understand how the programme was enabled and constrained by interactions between it and the context within which it was implemented, and whether these interactions produced any unintended consequences.

We adopted a multimethod, qualitative approach. We purposively selected^24^ five departments within a UK medical school that were completing applications for Athena SWAN awards. We aimed to represent a range in terms of departments' staff numbers, gender composition and stages of the Award application process.

The data were collected between January and April 2015 by DW, an experienced qualitative researcher with no prior connection to the HE or to the Athena SWAN programme, and involved observations of Athena SWAN SAT meetings in four of the case study departments. We were unable to undertake observations in Department 3 as this department did not hold any meetings during the duration of the fieldwork. The case study SATs were made up of between 6 and 17 members situated at all levels of seniority, from PhD students to Professors. SAT observations totalled 25 hours. One medical school-level and three HE-level Athena SWAN meetings were also observed to contextualise the departmental data. The data were collected through hand-written notes and were subsequently typed up. Observations were structured using an observation guide, which is summarised in box 1.

Box 1 **SAT observations topic guides**

- Who attends—gender and job title;
- Who does not attend (sends apologies)—gender and job title;
- Level of engagement of individual staff members in discussion;
- Who does Athena SWAN work;
- How participants position Athena SWAN and their experiences of it.

All Heads of Departments who were SAT members, SAT chairs and all SAT academic or administrative Leads in the five case study departments were invited by email and accepted to take part in interviews. In total 16 semistructured interviews were conducted, this included interviews with four key university personnel involved in the gender equity strategy. Interviews lasted from 24 to 54 min and were audio recorded.

Finally, we undertook four focus groups: two with postdoctoral researchers and two with lecturers and senior lecturers. Focus groups, rather than interviews, were undertaken in order to include the greatest number of participants, within the resource constraints of the study. Focus groups were segregated by the level of seniority to encourage participants to speak freely. All staff in these positions (153 postdoctoral researchers and 56 lecturers/senior lecturers) in the five case study departments were invited to take part by email. In total, 15 individuals (10 postdoctoral researchers and 5 lecturers/senior lecturers) participated in the 4 focus groups totalling 3 hours 21 min. Interview and focus group topics are documented in table 1.

**Table 1:** Interview and focus group topic guides

Interview topic guide	Focus group topic guide
Perceived impact of Athena SWAN.	Perceived impact of Athena SWAN.
Utility of Athena SWAN and for whom.	Perceived importance of gender equity and gender equity programmes.
Experiences and presence of Athena SWAN in the department.	Knowledge and understanding of Athena SWAN.
Knowledge of and perceived impact of funding incentive (if any).	Knowledge of and perceived impact of funding incentive (if any).
Anything inhibiting Athena SWAN work.	Experiences and presence of Athena SWAN initiatives.
Organisation and distribution of Athena SWAN work.	
Balancing Athena SWAN and other work.	

Data saturation was reached for interviews, which involved all eligible participants. A larger number of focus group participants may have yielded additional themes but could not be achieved since, as outlined above, we were unable to recruit further participants, despite contacting all eligible staff.

All interview and focus group audio recordings were transcribed verbatim. Transcripts and typed observation notes were uploaded to NVivo V.10. All data were analysed using thematic analysis.^25^ ^26^ Data were open coded for emerging themes with codes amended and consolidated iteratively throughout the process. Having read the transcripts, LC and DW met to discuss and agree the codes. They cross-checked each other's coding to ensure that codes matched data and that any counter examples within themes were coded and accounted for.

All interview and focus group participants gave written informed consent. Prior to the researcher's attendance at meetings, all SAT committee participants were provided with an information sheet about the study via email and informed that they could opt-out if they did not want their contributions to meetings recorded or included in the data. No one opted-out.

## Results

### Addressing gender inequity

Participants were overwhelmingly positive about the principles of Athena SWAN and reported positive outcomes from their implementation. The process of applying for Athena SWAN Awards was reported to have raised awareness of gender inequity and created social space to address problems:

It's been really useful in saying, “This is no longer acceptable; we have to do something about it.”— Interviewee 10, Professor, Female

There's a couple of sort of examples where people have been pulled up on things that they've said within [interview] panels because the person pulling them up has had the Unconscious Bias Training and actually, “Let's explore why you're saying this, is this anything to do with their capacity to do the job or is it something completely different?”— Interview 7, Department Manager, Female

Some participants reported that Athena SWAN galvanised and formalised the work their department was already doing to address gender inequity. Others reported that becoming involved in Athena SWAN highlighted to them and/or their colleagues, problems that needed to be addressed. For example:

And then reality dawned when I read the document and when I started to work with the team and I saw, as I said, I saw we didn't have, we had a very disjoined performance review, very dysfunctional promotions, approach to academic promotions and mentoring in the [Department]. So then I realised that actually yes this is, you know, this needs to be worked on.— Interviewee 5, Professor, Male

Participants from all levels of seniority recounted that the announcement that Silver Awards could be required for TROs to be eligible to re-apply for funding provided significant institutional drive to achieve Silver Athena SWAN awards. For example:

No [Department] that didn't have Silver could be part of the [TRO] renewal. That had really galvanised people […] Things had ticked over and ticked over and it wasn't until you had that galvanising right at the highest, you know, “This is real,” that everybody really put their shoulders to the wheel.— Interview 4, Professor, Female

### Factors undermining the potential impact of Athena SWAN principles

Factors potentially undermining the impact of Athena SWAN principles on gender inequity included the reproduction of gender inequity through a gendered distribution of Athena SWAN work, barriers to accessing Athena SWAN initiatives and limitations in Athena SWAN's scope. We discuss each of these factors below.

#### A gendered distribution of Athena SWAN work

Participant interviews and observations of SATs demonstrated that Athena SWAN applications involved a considerable burden of work. Many SAT participants reported that the awards application process increased their workload dramatically, leading to over-time and weekend work. For example:

The last two weeks of pulling this document together really has been exclusively Athena SWAN and probably to the detriment of some other, mostly my research, I would suggest, my PhD student has been somewhat neglected […]. It throws up some ridiculous scenarios. So, for example, I'm sitting at home on Saturday evening, writing in the Athena SWAN documents about work-life balance. And you think, “Something's not right here.”— Interviewee 11, Senior Lecturer, Female

Data collection on the SATs highlighted a gendered distribution of this workload: women were undertaking a disproportionate amount of Athena SWAN work. This was evident in a number of respects. First, women made up a larger proportion of SAT members. Of the 58 members on the five case study SATs, 20 were men (five of whom were Heads of Departments) and 38 (66%) were women. Munir *et al*'s^21^ study suggested that institutional champions and departmental champions were generally female. Therefore, together these findings indicate that Athena SWAN work is mainly led by and carried out by women.

The high proportion of women on SAT committees is not explained by departments' gender distribution. As table 2 demonstrates, across the case study departments, women's representation on SAT committees was disproportionately higher than their representation in departments. In Departments 1 and 3, their representation was substantially disproportionate.

**Table 2:** Percentage of women in departments and on SATs (2013–2014 departmental data)

Department	% of women in department	% of women on SAT
1	35	71
2	62	67
3	36	67
4	53	60
5	53	64

Observations of SATs revealed that while some men were actively engaged in each committee, in Departments 1, 2 and 4 women were undertaking the substantial work of pulling the document together. In Department 5, which was not yet the stage of writing the document, a woman was undertaking the principle task, which was data collection. Observations also highlighted the potential for a gendered working dynamic, in which women SAT members more actively volunteered for and delivered on tasks, compared to male colleagues.

The extract below from SAT field notes, provides an illustration of these dynamics in practice.

Box 2 **Fieldwork notes: SAT Observation extract**

SAT Meeting Attendance- Department 2

Participant 1 (P1, M)—Head of Department, Male

Participant 2 (P2, F)—Senior Lecturer, Female

Participant 3 (P3, F)—Senior Lecturer, Female

Participant 4 (P4, F)—Reader, Female

Participant 5 (P5, F)—Lecturer, Female

Participant 6 (P6, M)—Postdoc, Male

Participant 7 (P7, M)—Reader, Male

Extract:

P1 (M) directed the conversation to P7 (M) and asked what he thought of the application. P7 (M) asked what is most important. There was an awkward silence […]. He had not read the application yet. P1 (M) then asked P6 (M) what he thought and he said he's only read the first 10 pages but they seemed complete.

Throughout this meeting they were all rather animated except for P6 (M) and P7 (M)… P1 (M) was clearly engaged in the document and had read it carefully beforehand. Others such as P2 (F), P3 (F), P4 (F) and P5 (F) had printed, annotated copies of the document in front of them.

P1 (M) said that he thinks a half day [application writing workshop] is all we can stand. They suggested [date]. P1 (M) asked if anyone wouldn't mind doing it […]. P4 (F) was happy if it was a small group and they were going to actually do the work but couldn't do the proposed date and time. P5 (F) wasn't sure if she was available but would come if she was. P3 (F) was available, P1 (M) was available and so was P2 (F). P6 (M) and P7 (M) said nothing. P4 said she would rewrite the SAT part before the meeting and that everyone should comment on the full document in advance of this meeting.

In this extract, the male Head of Department is actively engaged and encourages other male members of the group to engage. However, in contrast to the female members, (with the exception of the Head of Department) the male members of the group have not read (or not fully read) the documents and do not volunteer for tasks. In the writing group meeting, which took place following this meeting, it was also observed that, in contrast to all other members, the two male members did not send comments on the document prior to the meeting.

In interviews, some SAT participants voiced concern that work to progress gender equity through Athena SWAN could simultaneously disadvantage individual women through the creation of additional labour that is not ‘counted’ towards career progression. For example:

We've asked for how [Athena SWAN work] will be acknowledged and it's been raised at faculty level also […] I flippantly say, “Well I could have written two papers in the time I've written this,” but it's not that flippant. I probably could have. So are we saying, “Well actually, the very document that is supposed to be helping to promote women is actually doing exactly the opposite because their workload is such that the women are doing it.”— Interviewee 11, Senior Lecturer, Female

Participants highlighted a similar paradox whereby the requirement to have women equally, rather than proportionately, represented on committees could create more work for women, compared to their male colleagues, and this work was not counted for promotion. Below a participant explains:

I wrote in my [Athena SWAN] document that I would make sure, we would strive to have the representation on decision-making committees that reflected the distribution of men and women in the department. And [the assessors] came back and said, “No, it should be fifty-fifty.” Well, you know, that just means a lot of poor women sitting on committees when again they should be doing their research and writing grants and things.— Interviewee 10, Professor, Female

#### Barriers to accessing Athena SWAN initiatives

In working towards Athena SWAN Awards, departments, and the institution as a whole, provided gender equity career development initiatives, including various mentoring and leadership schemes, aimed at addressing the disproportionate attrition of female postdoctoral researchers and lecturers/senior lecturers from the academic career ‘pipeline’. In order for these initiatives to be effective, however, staff must know about them and be able to avail of them.

Many participants who were involved in SATs were concerned that Athena SWAN committees should ensure that its initiatives are clearly meaningful and avoid becoming procedurally driven ‘tick-box’ exercises. This included concern that applying for an Athena SWAN Award should not come to be perceived as important simply for its financial incentive.

Our study highlights that some staff could not access Athena SWAN initiatives. Postdoctoral researchers who took part in the study were largely unaware of many Athena SWAN initiatives available to them and so had not accessed them. They reported that while they may have received information in emails, they suffered from email overload. The findings indicated that lecturers and senior lecturers were more aware of Athena SWAN initiatives because they were more integrated into departmental meetings than their postdoctoral colleagues.

Some postdoctoral researchers were also sceptical that the principal investigator (PI) they worked under would allow them time to attend Athena SWAN initiatives:

We get those [courses on personal/career development for postdoctoral researchers …] I know that at least my boss doesn't encourage us to go to those and we have to—yes we kind of have to push it, you know, ask the boss, you know, “Can I take a couple of hours off?” He's not one of those kind of guys who might say, you know, “Go and learn outside of the lab.”— Participant D, Postdoctoral researchers' focus group 1

#### Limitations in Athena SWAN's scope

Factors located outside of HEIs may undermine the impact of the Athena SWAN principles. These included institutional practices, national policies and societal norms.

First, the impact of Athena SWAN may be undermined by institutional practice in the TRO, which many HE staff are colocated in. Yet the TRO is neither the focus of Athena SWAN initiatives nor of assessment for Awards. For example, although HE Athena SWAN policy aims to ensure that meetings take place between 10 am and 16:00, in order to ensure that staff with caring responsibilities can attend, colocated staff still attended TRO meetings, which took place at 8.30am and after 16:00.

Similarly, different working practices between the HEI and thee hospital trust could undermine Athena SWAN initiatives for colocated staff:

[The clinical department had] their handover meetings, discussions about the clinical patients, at seven o'clock at night. Hell or high water. And that was because there was this idea that you, if you're going to do that specialty, you have to put these hours in […] you have to be there doing the same stuff that all the old men have always done in that discipline forever. And, of course, with that attitude, it doesn't provide any flexibility. And so if you are in a position where that doesn't suit you, your only option is to get out. And so you only get like-minded people pushing their way up.— Participant B, Postdoctoral researchers' focus group 2

This shows the limited scope of Athena SWAN when placed within the complex enmeshment of a TRO, HEI and hospital setting.

At the level of national policy, participants' experiences highlighted how family-friendly policies within the university could be undermined by national-level funding mechanisms. For example, one participant's experience illustrated how she felt she had to return to work early from maternity leave because the grant funding she had obtained did not provide maternity cover for her position. Her application to the HEI's own maternity fund was unsuccessful and so work was ‘building up’ in her absence. Similarly, some participants raised concerns that national funding structures were leading to the preponderance of temporary contracts (particularly at postdoctoral level) and pressure to publish, even while on maternity leave. These funding mechanisms were seen as gender biased since they disproportionately affected women:

I think when I've seen it work for women to really move up and become independent researchers is when they've had a really supportive boss. But they've still had to be publishing while they've been on maternity leave and things like that. And I think that comes from a grant government level, because whilst you're still allowed to put you've had a break for family reasons, and whichever, whilst they take that into consideration, you still are seen to have to be publishing while you're on maternity leave. And I think that's probably one big reason [for gender inequity in academic medicine].— Participant D, Postdoctoral researchers' focus group 2

Finally, some participants were frustrated that, as they perceived it, Athena SWAN principles emphasise the need to support women through family-friendly provision, without challenging the underlying societal problem of a gendered division of caring labour. For example:

Sometimes I think the emphasis [in Athena SWAN] is too much on the mother needing to have flexibility, why can't it be more acceptable for the men to take more of the role and have the flexibility?— Participant C, Lecturers'/ senior lecturers' focus group 1

Even if it was pitched at, “Men, you can get childcare support,” you know, and that, even if their wife or partner or girlfriend or whatever isn't even working in science, if they could see that as, you know, it's moving out of the system of, you know, it doesn't have to just be [women].— Participant D, Postdoctoral researchers' focus group 2

In this respect, the perceived emphasis on supporting women through family-friendly policies was seen by some to reinforce the assumption of women's role in undertaking a disproportionate amount of caring work.

## Discussion

Gender equity programmes, such as Athena SWAN, represent an effort to address the international problem of the under-representation of women in academic medicine and biomedical science. Adopting a realist evaluation approach, this study sought to understand how implementation of Athena SWAN principles was enabled and constrained by interactions between the programme and its context and whether these interactions produced any unintended consequences. Our findings raise important considerations for such programmes, which we discuss below.

Although the number of women in employment in academic medicine has increased since Athena SWAN Awards were introduced, econometric analysis^20^ has not found that this increase was due to Athena SWAN. However, more time, and perhaps a larger number of medical schools, which would improve the sensitivity of the measure, may be required to demonstrate this. Other research^21^ has suggested that women report a positive impact of Athena SWAN on intermediate indicators of impact, including their self-confidence, visibility and leadership skills. Participants in our study were overwhelmingly positive about Athena SWAN principles and our research contributes an understanding of the mechanisms behind Athena SWAN's impact, illustrating how Athena SWAN principles can drive the gender equity agenda by highlighting problematic practice and empowering staff to address it. Our study also provides substantiation to claims^20^ ^21^ that linking this gender equity initiative to funding has been an important support factor in encouraging institutional backing to achieve awards.

Furthermore, our research makes a unique contribution by drawing attention to factors that may undermine the impact of Athena SWAN on gender equity. Gender inequity is typically viewed as a product of a bygone era, with work in the present assumed to be progressive. Yet our findings highlight how gender inequity can be unintentionally reproduced in the enactment of initiatives. Women in all our case study departments were undertaking a disproportionate burden of Athena SWAN work, which was not counted towards career progression. This reproduction of gendered working creates a paradox in which the enactment of Athena SWAN has the potential to unintentionally reinforce inequity, since women's participation in the scheme may disadvantage their individual career progression.

This study has identified the potential for a gendered distribution of Athena SWAN work to occur but it cannot empirically verify why such a distribution emerged. A simple explanation might be that, as the under-represented gender, women felt more personal interest in becoming involved in a programme for change and the programme itself does not provide a mechanism to ensure that this work is evenly distributed. However, it should also be noted that the gendered distribution of Athena SWAN work is in keeping with a wider trend of women undertaking a disproportionate burden of work that is undervalued in monetary terms. Despite increased female participation in the labour market, women as a group continue to do a disproportionate amount of unpaid household and caring work, compared to their male partners.^27^ ^28^ In HE specifically, it has been found that women in UK and US universities are doing more hours of teaching, especially at the lowest levels (introductory courses rather than PhD work), more pastoral care, more administration and more committee work, compared to male colleagues; women are doing less research and publication work, which are usually the key criteria for promotion.^29^ In this sense, it could be argued that it is the valuing of Athena SWAN work (which, at least in the HEI studied here, does not count towards promotion) that creates the career disadvantage for women involved in it, rather than women's involvement per se. Regardless, the unequal distribution of work could be addressed by mandating proportional representation of men and women on SATs and by ‘counting’ this work for promotion.

The Athena SWAN evaluation process adopts an audit approach to assessing HEIs for awards, characterised by account giving and checking.^30^ ^31^ Given that research in other areas has shown how audit processes can become ends in themselves or, as Power^30^ describes them, ‘rituals of verification’, there is a need to avoid a process-driven approach. This involves ensuring that Athena SWAN initiatives are clearly supported to achieve outcomes on gender equity. Our finding that there are barriers to postdoctoral researchers accessing some Athena SWAN initiatives suggests that this is not always the case. This may explain why Munir *et al*^21^ found that research staff responding to their survey rated the impact of Athena SWAN on them lower than academic staff. In part this points to the need to consider how initiatives are communicated to staff to ensure they are aware of them. However, the fact that more junior research staff reported that their supervising colleagues may not allow them to attend Athena SWAN initiatives highlights the need for widespread support throughout an institution in order for initiatives, like Athena SWAN, to have the potential to work.^32^

Furthermore, this research highlights how gender equity initiatives may be undermined by wider factors.^32–34^ We identify factors at institutional, national and societal levels. At the institutional level, our finding that practice in colocated TROs and hospitals can undermine the impact of gender equity initiatives in HEIs lends support to proposals to assess and address gender inequity in TROs, rather than focusing narrowly on practice in HEIs.^13^ At the national level, the data also highlight how family-friendly policies, introduced through Athena SWAN in order to support women's employment, can be undermined by national level policies. Some progress seems to have been made recently in addressing the absence of maternity/parental cover in some UK funding streams,^35^ although gaps remain.^36^ However, the prevalence of temporary contracts and pressure to publish, even during maternity leave, remain. Such policies may be particularly unattractive to women who have or intend to have children.^32^ ^34^ In this sense, our study highlights the importance of a joined-up approach to addressing gender inequity, linking institutional and national policy. The findings call into question whether action at the HE level is sufficient to address gender inequity in academic medicine. One solution in this regard might be for the Athena SWAN programme and its member HEIs to identify wider barriers to gender equity and to advocate for national policies to support the programme's aims.

Finally, societal-level norms may undermine impact. The assumption that women should undertake a disproportionate amount of caring work is a key contribution to gender inequity.^8^ ^37^ Athena SWAN articulates family-friendly policies as supportive of working mothers and fathers. However, as our participants highlighted, by focusing on championing family-friendly policies within an initiative to support women's representation in the labour force, Athena SWAN may reinforce the perception that it is women's place to undertake caring work. In this way, the programme may unintentionally contribute to a key mechanism supporting unequal gendered participation in academic medicine.^34^ Indeed, previous research has suggested that family-friendly policies can be perceived as enabling employees with family commitments to work at the margins, but that they seldom challenge traditional patterns of work as the norm and the ideal.^34^ ^38–40^ As participants suggested, initiatives could, for example, challenge gendered norms by shifting the focus to encourage and support male employees to avail of the opportunity to share parental leave. This would seem a worthwhile focus particularly since it has been found that family-friendly policies to support men to be more involved in caring responsibilities are often undermined by work place cultures.^33^ ^41^

## Strengths and limitations

The study's qualitative focus on five departments within one institution identifies new issues, including how Athena SWAN can address gender inequity, factors that may impede its impact and unintended programme consequences. We do not quantitatively assess the national or international prevalence of these issues. Nor do we claim that our findings are exhaustive: further research could helpfully develop our work.

As noted in the ‘Methods’ section, no meetings took place in Department 3 and so the interview data could not be triangulated. However, the interviews in this department identified the same themes that were common across the departments and our analysis of the gender composition of the department revealed the same gender imbalance on the Athena SWAN committee. Therefore, although we could not triangulate these data through observation, our data suggest that the same themes presented in this department as in the others.

## Conclusions

The underrepresentation of women in senior academic medical positions has been internationally identified as a problem to be addressed. This study contributes to the literature on gender equity in academic medicine by illustrating how gender equity programmes have the potential to contribute to addressing gender inequity, and are seen by staff as important and useful. However, gender equity programmes can also reproduce and reinforce inequity in their enactment. Further, we illustrate how programmes can be undermined where staff cannot avail of initiatives and where wider institutional practice, national policies or societal norms undermine its effects.

This is the first study to investigate how Athena SWAN interacts with the contexts it is introduced into. Further studies are needed. Subsequent research could develop our findings by identifying additional factors that may constrain gender equity programme effects as well as those that might support them. A comparative case study approach would be particularly useful in order to build our understanding of how programme mechanisms interact with contexts. Further research could also investigate whether the recent refocusing of the Athena SWAN principles on gender parity has impacted perceptions or experiences of the programme.
